# Stability-Indicating Analytical Approach for Stability Evaluation of Lactoferrin

**DOI:** 10.3390/pharmaceutics13071065

**Published:** 2021-07-11

**Authors:** Nika Osel, Timeja Planinšek Parfant, Albin Kristl, Robert Roškar

**Affiliations:** Faculty of Pharmacy, University of Ljubljana, Aškerčeva Cesta 7, 1000 Ljubljana, Slovenia; nika.osel@ffa.uni-lj.si (N.O.); timeja.planinsek.parfant@ffa.uni-lj.si (T.P.P.); albin.kristl@ffa.uni-lj.si (A.K.)

**Keywords:** lactoferrin, stability, HPLC-UV, spectroscopic methods, stability-indicating approach, proteins, quality control

## Abstract

Lactoferrin is a multifunctional iron-binding glycoprotein in milk. Due to its potential for the treatment of various diseases, interest in products containing lactoferrin is increasing. However, as a protein, it is prone to degradation, which critically affects the quality of products. Therefore, the main purpose of our work was to develop a stability-indicating analytical approach for stability evaluation of lactoferrin. We were focused on two complementary methods: reversed-phase and size-exclusion chromatography. The stability-indicating nature of the selected methods was confirmed. They were successfully validated by following the ICH guidelines and applied to preliminary lactoferrin stability studies. Up to three degradation products, as well as aggregates and fragments of lactoferrin, were detected in various samples using complementary reversed-phase and size-exclusion chromatographic methods. The analytical approach was additionally extended with three spectroscopic techniques (absorbance, intrinsic fluorescence, and bicinchoninic acid method), which may provide valuable complementary information in some cases. The presented analytical approach allows the stability evaluation of lactoferrin in various samples, including the ability to detect differences in its degradation mechanisms. Furthermore, it has the potential to be used for the quality control of products containing lactoferrin.

## 1. Introduction

Lactoferrin (Lf) is an 80-kDa non-hemic iron-binding basic (isoelectric point of 8.4–9.0) globular glycoprotein from the transferrin family, which is found in various secretory fluids [1,2,3]. Its polypeptide chain consists of about 700 amino acids folded into two symmetrical lobes. Each lobe has a high-affinity binding site for an iron ion, which is bound together with a carbonate ion [1]. Lf is the main iron-binding protein in milk with antimicrobial, immunomodulatory, antioxidant, anticancer, and many other biological functions. The peptides resulting from its proteolytic degradation also possess some of the functions of the intact protein [1,3]. It is the second most abundant whey protein in human milk and is also present in the milk of most mammals. Due to its positive effects, it is commercially purified from milk and added as an active ingredient to infant milk formulas, food supplements, food products, beverages, cosmetics, and pet foods [1,2,3]. Several clinical studies showed its potential to be used as a primary or adjuvant agent in the treatment of infections, cancers, sepsis, and other diseases [3,4,5].

A literature review showed a lack of stability data for Lf, as only a few studies investigated Lf stability in various media and formulations. Lf concentration in human milk significantly decreased after three months at −18 to −20 °C, but was stable for five days in the refrigerator [6]. The integrity and concentration of Lf in yogurt remained constant throughout 28 days of storage [7]. In the liquid formulation of Lf, the addition of arginine had a stronger stabilizing effect than the addition of human serum albumin [8]. Lf in liposomes was completely degraded after six months, whereas almost 30% of intact Lf remained in the formulation of solid lipid particles [9]. The degree of iron saturation had a significant effect on the thermal stability of Lf in aqueous solutions when heating between 50 and 90 °C and also when drying it at 70 and 95 °C [10,11]. The most important factors affecting the stability of Lf are temperature, pH, ionic strength, high pressure, and the presence of polysaccharides or other proteins [12]. As a serine protease, Lf is also prone to auto-proteolytic degradation [13].

The evaluation of protein stability demands a complex analytical approach [14]. In the above mentioned studies, the enzyme-linked immunosorbent assay (ELISA) [6,7] and high-performance liquid chromatography (HPLC) [8,9,11] were the most commonly used techniques for the evaluation of the Lf stability. ELISA is a very specific, sensitive, and high-throughput technique. However, its disadvantages compared to HPLC methods are a very narrow linearity range, higher variability, and high costs, which limit its routine use [15,16]. HPLC is one of the key techniques for a simple and low-cost protein characterization [14,16]. Size-exclusion HPLC (SEC-HPLC) is mainly used for the evaluation of Lf aggregation, integrity, and purity [10,17,18]. Reversed-phase HPLC (RP-HPLC) methods are suitable for the qualitative and quantitative analysis, of various Lf samples. They are also used for the detection of changes in protein integrity such as degradation, decarboxylation, and deamidation [8,16,17]. Recently, an analytical ion-exchange chromatographic method was developed for the determination of Lf content in various samples although this technique is usually used to purify Lf from milk [17]. In addition to ELISA and HPLC, sodium dodecyl sulfate-polyacrylamide gel electrophoresis (SDS-PAGE) is commonly complementing other techniques used in the Lf stability studies [7,8,9,10]. Different spectroscopic techniques are also used for the evaluation of thermodynamic stability of Lf. Changes in Lf structure can be evaluated by fluorescence measurements [19,20,21]. UV spectroscopy is used for the determination of denaturation temperature [20,21], as well as for the determination of Lf content [22] and iron saturation [10,23].

Using stability-indicating methods is crucial in stability studies to accurately measure the amount of active ingredients without interference from degradation products, impurities, and excipients [24]. To the best of our knowledge, the stability-indicating nature of HPLC methods used for the determination of Lf has not been demonstrated. Therefore, the main purpose of this work was to develop a stability-indicating analytical approach comprised of complementary RP-HPLC and SEC-HPLC methods to be used for stability evaluation of Lf in preformulation studies, as well as for quality control in final products. In addition, we considered the feasibility of using fast, simple and accessible spectroscopic techniques for stability testing of Lf. Finally, the adequacy of the validated stability-indicating analytical approach in preliminary stability tests, as well as in quality control testing of products containing Lf was investigated.

## 2. Materials and Methods

### 2.1. Reagents and Chemicals

Standards of lactoferrin from bovine colostrum (≥85%), type I α-lactalbumin from bovine milk (≥85%), β-lactoglobulin from bovine milk (≥90%), and lactoperoxidase from bovine milk (≥200 units/mg protein) were purchased from Sigma-Aldrich (St. Louis, MO, USA). HPLC-grade acetonitrile (ACN) and 30% H_2_O_2_ solution were purchased from Honeywell (Seelze, Germany). Disodium hydrogen phosphate anhydrous, hydrochloric acid (HCl) for 1 M solution, phosphoric acid (85%), sodium chloride (NaCl), sodium dihydrogen phosphate monohydrate, sodium hydroxide (NaOH) for 1 M solution, trifluoroacetic acid (TFA), and Millex^®^-GV PVDF filters (0.22 µm) were purchased from Merck (Darmstadt, Germany). A BCA^TM^ Protein Assay Kit was purchased from Thermo Scientific (Rockford, IL, USA). High purity Milli-Q water was obtained through a Milli-Q A10 Advantage water purification system (Millipore Corporation, Bedford, MA, USA). Different lots of solid Lf isolate samples (1–3; A-value of 3.4–5.2% [21]), as well as liquid Lf isolate samples (elutions E1, E3, and concentrated E3) were obtained from ARHEL d.o.o. (Ljubljana, Slovenia). Three commercially available capsules containing Lf were purchased from a Slovenian market). Additional data about samples can be found in Table A1.

### 2.2. Reversed-Phase HPLC Method

The RP-HPLC analysis was performed on Agilent 1100/1200 series HPLC systems (Agilent Technologies, Santa Clara, CA, USA), equipped with diode array or variable wavelength detector and ChemStation data acquisition program. Detection was carried out at 280 nm. Separation was performed on a BioZen™ Intact XB-C8 column (150 × 4.6 mm, 3.6 μm; Phenomenex, Torrance, CA, USA) at 30 °C by gradient elution using 0.1% TFA in water (A) and 0.1% TFA in ACN (B) as a mobile phase at a flow rate of 1.0 mL/min with the following gradient program: 0.0–3.0 min 20.0–36.0% B, 3.0–5.0 min 36.0% B, 5.0–5.1 min 36.0–39.0% B, 5.1–6.5 min 39.0% B, 6.5–9.8 min 39.0–50.0% B, 9.8–12.0 min 50.0% B, 12.0–12.1 min 50.0–20.0% B, and 12.1–16.0 min 20.0% B. The injection volume was 20 μL.

The optimized chromatographic conditions for other tested RP-HPLC columns are presented below. Separation on BIOShell™ A400 Protein C18 column (100 × 2.1 mm, 3.4 μm; Sigma-Aldrich, St. Louis, MO, USA) using 0.1% TFA in water (A) and ACN (B) as a mobile phase at a flow rate of 0.8 mL/min and column temperature of 40 °C had the following gradient program: 0.0–3.0 min 20.0–38.1% B, 3.0–5.5 min 38.1% B, 5.5–5.6 min 38.1–41.0% B, 5.6–7.0 min 41.0% B, 7.0–9.7 min 41.0–50.0% B, 9.7–12.0 min 50.0% B, 12.0–12.1 min 50.0–20.0% B, and 12.1–16.0 min 20.0% B. Separation on a Vydac^®^ Protein C4 #197 column (250 × 4.6 mm, 5 μm; W. R. Grace & Co.-Conn., Columbia, MD, USA) using 0.03% TFA and 0.05 M NaCl in water (A) and ACN (B) as a mobile phase at a flow rate of 1.0 mL/min and column temperature of 30 °C had the following gradient program: 0.0–3.0 min 20.0–37.1% B, 3.0–5.0 min 37.1% B, 5.0–5.1 min 37.1–40.0% B, 5.1–6.5 min 40.0% B, 6.5–9.5 min 40.0–50.0% B, 9.5–12.0 min 50.0% B, 12.0–12.1 min 50.0–20.0% B, and 12.1–16.0 min 20.0% B. Separation on a Zorbax 300SB-C3 column (150 × 2.1 mm, 5 μm; Agilent Technologies, Santa Clara, CA, USA) using 0.1% TFA in water (A) and ACN (B) as a mobile phase at a flow rate of 1.0 mL/min and column temperature of 35 °C had the following gradient program: 0.0–3.0 min 20.0–37.0% B, 3.0–5.0 min 37.0% B, 5.0–5.1 min 37.0–40.0% B, 5.1–6.5 min 40.0% B, 6.5–9.5 min 40.0–50.0% B, 9.5–12.0 min 50.0% B, 12.0–12.1 min 50.0–20.0% B, and 12.1–16.0 min 20.0% B. SecurityGuard™ C18 (4 × 3.0 mm; Phenomenex, Torrance, CA, USA) guard column was used along with selected columns.

### 2.3. Size-Exclusion HPLC Method

Agilent 1100/1200 series HPLC systems (Agilent Technologies, Santa Clara, CA, USA), equipped with diode array or variable wavelength detector and ChemStation data acquisition program were used. Detection was carried out at 280 nm. Separation was performed on an XBridge^®^ Protein BEH SEC 200 Ǻ column (150 × 7.8 mm, 3.5 μm; Waters, Milford, MA, USA) at 25 °C using 0.2 M sodium phosphate buffer (pH 6.8) containing 0.1 M NaCl as a mobile phase at a flow rate of 0.86 mL/min. The injection volume was 10 μL. Another tested SEC-HPLC column was a BioSep™-SEC-S 2000 column (300 × 7.8 mm, 5 μm; Phenomenex, Torrance, CA, USA). Isocratic elution was performed at 25 °C using 0.05 M sodium phosphate buffer (pH 7.0) containing 0.5 M NaCl as a mobile phase at a flow rate of 0.8 mL/min. The injection volume was 20 μL. SecurityGuard™ C18 (4 × 3.0 mm; Phenomenex, Torrance, CA, USA) guard column was used along with the selected columns.

### 2.4. Spectroscopic Methods

All spectroscopic measurements were carried out on a Safire2 microplate reader (Tecan, Männedorf, Switzerland). UV-STAR^®^ transparent 96-well microplates (Greiner Bio-One, Kremsmünster, Austria) were used to obtain UV absorption spectra and absorbance at 280 nm (A_280nm_).

Total protein concentration was determined by the bicinchoninic acid method (BCA) using the BCA™ Protein Assay Kit. The absorbance measurements were carried out at 562 nm and the samples were prepared as described in the user manual in the chapter Microplate Procedure (sample to working reagent ratio = 1:8) [25]. White 96-well microplates with transparent flat bottom were used for these measurements (Greiner Bio-One, Kremsmünster, Austria).

Intrinsic fluorescence measurements (FLD) were performed on Nunc™ 96-well black microplates (Thermo Scientific, Rockford, IL, USA). The excitation wavelength and the emission wavelength were set at 285 nm and 350 nm, respectively. The excitation and emission slit wavelengths were both set at 9 nm.

### 2.5. Method Validation

The optimized RP-HPLC (on BioZen™ Intact XB-C8 column) and SEC-HPLC (on XBridge^®^ Protein BEH SEC 200 Ǻ column) methods were validated according to the ICH Q2 (R1) guidelines [26]. Specificity, linearity, accuracy, precision, detection and quantification limits and sample stability were evaluated. Validation of spectroscopic methods was performed to a lesser extent than chromatographic methods.

#### 2.5.1. Specificity

Specificity was evaluated by comparing the chromatograms of the Lf isolate solution, compounds used in the sample preparation (H_2_O_2_, NaOH, HCl, etc.), commercial products containing Lf, and standard solutions of the most common whey proteins (α-lactalbumin, β-lactoglobulin, lactoperoxidase). The HPLC chromatograms were visually inspected for interfering peaks at the retention time of Lf. Specificity of all methods was also evaluated by forced degradation samples.

#### 2.5.2. Calibration Curve and Sensitivity

Eight calibration standards were used for the calibration curve. The corresponding equations and determination coefficients (R^2^) were determined for different models (linear, weighted linear, quadratic) based on the least-square regression. The acceptance limit was set at R^2^ > 0.999. The model with the highest R^2^ and the lowest calibration curve residuals was chosen. LOD and LOQ were defined as concentrations corresponding to the signal-to-noise ratio multiplied by 3 and 10, respectively.

#### 2.5.3. Accuracy and Precision

Intra- and inter-day accuracy and precision (repeatability, intermediate precision) of both chromatographic methods were evaluated during three validation days. Three replicate analyses of quality control samples covering the whole analytical range were performed. Injection repeatability was evaluated by re-injecting the medium concentration quality control sample six times. Precision, calculated as relative standard deviation (RSD), should be less than 5% and less than 2% for injection repeatability. Accuracy was defined as the ratio (%) between the determined and theoretical concentration of Lf. It should be within 95 and 105%. Similarly, the intra-day accuracy and precision of spectroscopic methods were evaluated.

Method recovery (RP-HPLC, A_280nm_, and BCA method) was evaluated by separately analyzing the spiked preparations, non-spiked preparations, and standard solutions containing the added Lf amounts. Average recoveries were calculated by the following Equation (1):

(1) $$\mathrm{Recovery}\;\left[\%\right]=100\;\times \;\frac{\mathrm{concentration}\;\mathrm{in}\;\mathrm{spiked}\;\mathrm{sample}-\mathrm{concentration}\;\mathrm{in}\;\mathrm{unspiked}\;\mathrm{sample}}{\mathrm{added}\;\mathrm{concentration}}$$

#### 2.5.4. Sample Stability

Sample stability in the autosampler at 25 °C was evaluated using the RP-HPLC method. One parallel of each quality control sample was re-analyzed after 24 and 48 h. The Lf concentration at a certain time point was compared to the one at time point zero. The ratio expressed in percent should be within 95 and 105%.

### 2.6. Sample Preparation Protocols

All sample solutions were filtered before analysis using low-retaining PVDF filters. The suitability of filters was analytically evaluated. No differences between filtered and non-filtered samples were observed.

#### 2.6.1. Procedure for Forced Degradation Study of Lactoferrin

Lf stress samples were prepared by dissolving the solid Lf isolate sample 1 in Milli-Q water (c = 1.0 mg/mL). The solution was filtered and the procedure was continued as stated in Table 1. Control samples of Lf in Milli-Q water were stored at room temperature. Blank samples were also prepared by substituting the Lf sample with Milli-Q water. Stress conditions and time points were adjusted by preliminary analyses. The targeted extent of Lf degradation was between 10 and 20%. The same samples were also used for the evaluation of spectroscopic methods. These samples were diluted with Milli-Q water two-fold, three-fold, and ten-fold for the A_280nm_, BCA, and FLD method, respectively.

#### 2.6.2. Preparation of Solutions for Method Validation

For the specificity evaluation of both chromatographic methods, Lf stress samples were prepared as described in Section 2.6.1. Additionally, specificity was evaluated by analyzing standard solutions of other whey proteins. 2.0 mg of each whey protein standard was accurately weighed and dissolved with 2.0 mL of Milli-Q water in a volumetric flask.

The standard Lf solution (*c* = 5.0 mg/mL) was prepared by accurately weighed 10.0 mg of Lf standard and dissolved with Milli-Q water in a 2.0 mL volumetric flask. For both chromatographic methods and A_280nm_ method this standard solution was diluted with Milli-Q to obtain eight calibration standards of the following concentrations: 0.05, 0.10, 0.25, 0.50, 1.0, 1.75, 2.5, and 5.0 mg/mL. For the FLD method these calibration standards were 10-fold diluted with Milli-Q water. For the BCA method the same standard solutions were used and appropriately diluted to obtain calibration standards with the following concentrations: 0.025, 0.050, 0.100, 0.250, 0.500, 0.750, 1.0, and 2.0 mg/mL.

20.0 mg of Lf standard was accurately weighed and dissolved with 5.0 mL of Milli-Q water in a volumetric flask. This solution was used as the quality control samples at high concentration (QC_h_, 4.0 mg/mL). The QC_h_ sample was diluted with Milli-Q water to obtain quality control samples at low (QC_l_, 0.15 mg/mL) and medium concentration (QC_m_, 0.75 mg/mL). The same quality control samples as for both chromatographic methods were also used for the evaluation of the A_280nm_ method. For the FLD and BCA method, these samples were 10-fold and 2.5-fold diluted with Milli-Q, respectively. Three parallels of quality control samples were prepared each day of validation.

Method recovery was determined by spiking the products containing Lf with approximately the same amount of the Lf standard as contained in the preparations (see Section 2.6.3.). Recovery samples were prepared in triplicate. For the BCA method, the spiked samples were diluted with Milli-Q water two-fold.

#### 2.6.3. Evaluation of Lactoferrin Content in Products

The content of Lf in three commercially available products was evaluated by RP-HPLC, A_280nm_, and BCA method. The content of each hard capsule was dissolved in Milli-Q water in a volumetric flask to obtain Lf concentration of about 1.0 mg/mL according to the labeled content (Table A1). Samples were stirred on a magnetic stirrer for 2 h. Four parallels of each product sample were analyzed.

#### 2.6.4. Stability of Lactoferrin at High Temperatures

A total of 50.0 mg of solid Lf isolate sample 1 was accurately weighed and dissolved with 50.0 mL of Milli-Q water in a volumetric flask (c = 1.0 mg/mL). Additionally, a sample of Jarrow Formulas capsules with Lf concentration of about 1.0 mg/mL was prepared as described in Section 2.6.3. All solutions were filtered and exposed to elevated temperatures in heat chambers. The stability of Lf was evaluated at four temperatures: 50, 60, 70, and 80 °C. The stability of Lf in commercial capsules was evaluated only at 60 °C. The samples were sampled at pre-defined time points and cooled down to room temperature and directly analyzed. Opaque/cloudy sample solutions were filtered again before analysis. All samples were prepared in duplicate. Additionally, the stability of Lf in three lots of solid Lf isolate samples was evaluated at 50 °C. Samples were prepared as described above in triplicate.

#### 2.6.5. Stability of Lactoferrin in Liquid Elution Samples and Effect of Lactoferrin Concentration on Its Stability

Liquid Lf elution sample E1 (c = 0.55 mg/mL) was stored at controlled room temperature (up to 25 °C) and analyzed at pre-defined time points (up to 7 days). Liquid Lf elution samples E3 (c = 1.9 mg/mL) and concentrated E3 (c = 30.5 mg/mL) were stored at controlled room temperature (up to 25 °C), in a refrigerator (4 °C), and in a freezer (−20 °C). Both samples were analyzed at pre-defined time points (up to 28 days). Each E3 and concentrated E3 sample was 10-fold and 50-fold diluted with Milli-Q water, respectively. Sample E1 was directly analyzed. Samples E3 and concentrated E3 were prepared in triplicate for each storage condition, whereas only one parallel of sample E1 was analyzed.

The effect of Lf concentration on its stability was also evaluated. Solid Lf isolate sample 3 was accurately weighed and dissolved with Milli-Q water in a volumetric flask to obtain the following concentrations: 0.55, 1.9, 10.0, and 30.5 mg/mL. Samples were stored at controlled room temperature (up to 25 °C) and analyzed at pre-defined time points (up to 28 days). 10.0 mg/mL and 30.5 mg/mL samples were 10-fold and 30-fold diluted with Milli-Q water, respectively. All samples were prepared in triplicate.

### 2.7. Degradation Kinetics

The mean values, standard deviations, relative standard deviations, and determination coefficients were calculated using MS Excel (Excel version 2010). MS Excel and IBM^®^ SPSS^®^ Statistics 21 software were used for calibration curve data analysis. Zero, first and second-order kinetics were fitted to Lf degradation by using the least-square regression. Fitted models were evaluated by using determination coefficients (R^2^) and the model with the highest R^2^ was selected. The rate constants were determined based on the most adequate model. Quantitative dependence between the rate constant and the absolute temperature was calculated by the Arrhenius equation (Equation (2)), where k is the rate constant, T is the absolute temperature, R is the universal gas constant, A is the frequency factor, and Ea is the activation energy for the reaction.

(2) $${k=\mathrm{Ae}}^{\frac{-\mathrm{Ea}}{\mathrm{RT}}}\;$$

## 3. Results

### 3.1. Reversed-Phase HPLC Method Development and Optimization

**Figure A1:**
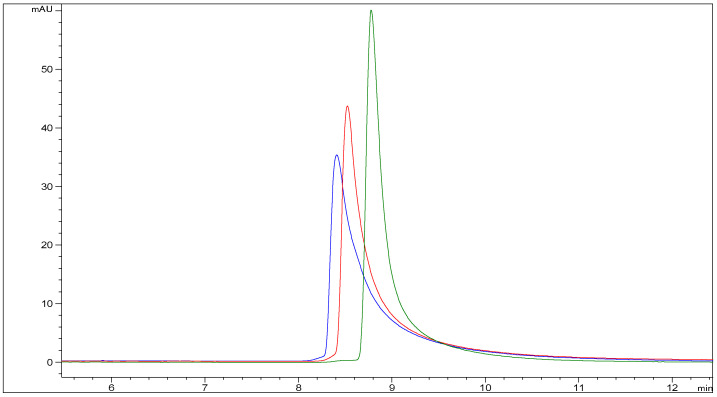
The effect of TFA concentration in the mobile phase on Lf peak shape: −− 0.03% TFA, −− 0.05% TFA, and −− 0.1% TFA. Separation was performed on Vydac^®^ Protein C4 #197 column at 30 °C using TFA in water (A) and acetonitrile (B) as a mobile phase at a flow rate of 1.0 mL/min and the following gradient program: 0.0–10.0 min 20.0–60.0% B, 10.0–15.0 min 60.0% B, 15.0–16.0 min 60.0–20.0% B, 16.0–20.0 min 20% B.

**Figure A2:**
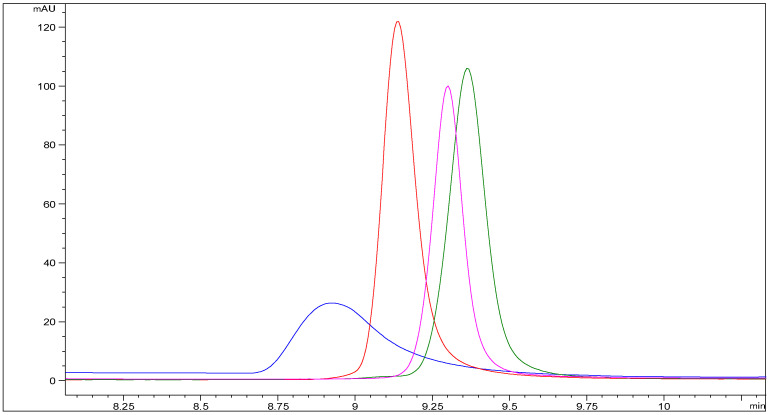
The effect of NaCl concentration in the mobile phase on Lf peak shape (RP column Vydac^®^ Protein C4 #197): −− 0.0 M NaCl, −− 0.05 M NaCl, −− 0.25 M NaCl, and −− 0.5 M NaCl. Separation was performed on Vydac^®^ Protein C4 #197 column at 30 °C using 0.03%TFA and NaCl in water (A) and acetonitrile (B) as a mobile phase at a flow rate of 1.0 mL/min. The gradient program is described in Section 2.2.

**Figure A3:**
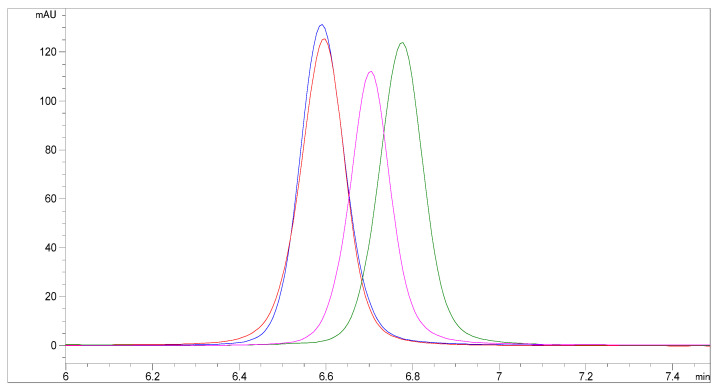
The effect of NaCl concentration in the mobile phase on Lf peak shape: −− 0.0 M NaCl, −− 0.05 M NaCl, −− 0.25 M NaCl, and −− 0.5 M NaCl. Separation was performed on Zorbax 300SB-C3 column at 35 °C using 0.1% TFA and NaCl in water (A) and acetonitrile (B) as a mobile phase at a flow rate of 1.0 mL/min. The gradient program is described in Section 2.2.

RP-HPLC method development was quite challenging due to the complexity of the separation mechanism. Four RP-HPLC columns intended for protein analysis were tested. The selected columns differed in stationary phase chemistry, dimensions, particle sizes, and particle porosity. The first method development step was the Lf peak shape optimization on each column using the Lf isolate sample. The HPLC conditions were optimized for the mobile phase composition, gradient program, flow rate, and column temperature for each selected column. Only one chromatographic condition was optimized at a time. A mobile phase composed of TFA in water and ACN was used for the gradient elution of Lf. A higher concentration of TFA (0.1% versus 0.05% and 0.03%) resulted in a sharper chromatographic peak of Lf (Figure A1). The addition of NaCl to the mobile phase was crucial for the older type of traditional porous column (Vydac^®^ Protein C4 #197). It resulted in a sharper Lf peak and better resolution between Lf and impurity peaks in the sample. Three different NaCl concentrations (0.05, 0.25 and 0.5 M) were tested. The lowest NaCl concentration was sufficient for an improved peak shape on Vydac^®^ Protein C4 #197 column (Figure A2). However, for the tested columns Zorbax SB-300 C3, Biozen™ Intact XB-C8, and BIOShell™ A400 Protein C18, the addition of NaCl was non-essential or even worsened the Lf peak shape (Figure A3). The gradient program was optimized for each selected column using a binary mobile phase system. Interestingly, the percent of organic solvent needed for the elution of Lf was between 36 and 39% regardless of the stationary phase chemistry (C3–C18).

**Figure A4:**
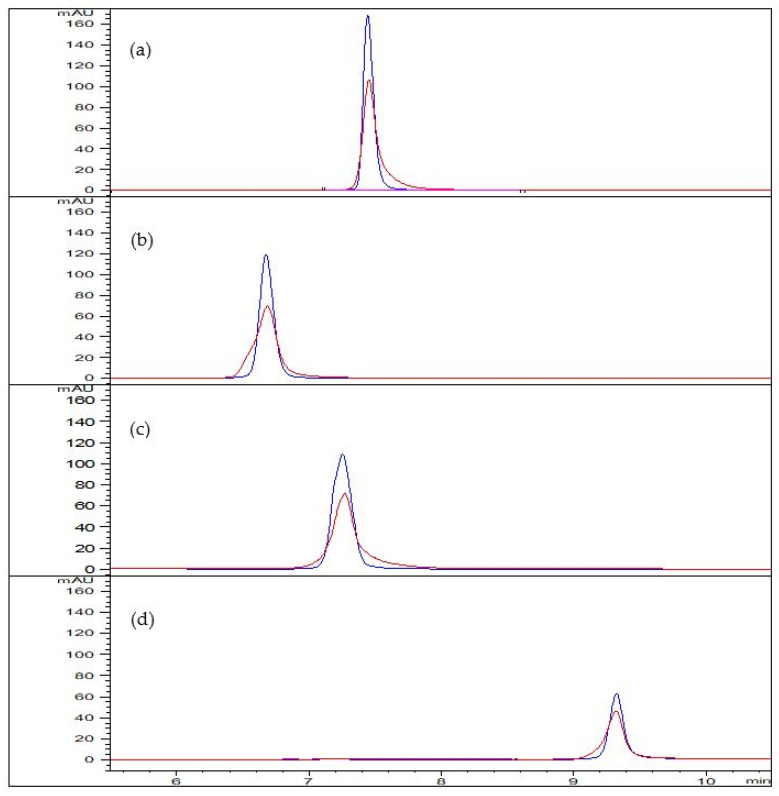
Comparison of optimized RP-HPLC methods on selected columns: (**a**) BioZen™ Intact XB-C8 column, (**b**) Zorbax 300SB-C3 column, (**c**) BIOShell™ A400 Protein C18 column, and (**d**) Vydac^®^ Protein C4 #197 column; −− control sample and −− alkaline stress sample.

In the second method development step, Lf stress samples were introduced as they are crucial for the stability-indicating method development. The goal was to achieve the separation between Lf and its degradation products. However, the degradation products in Lf stress samples could not be detected by using any of these columns. The chromatographic peak for Lf broadened regardless of the column used, which typically occurred in alkaline stress samples (Figure A4). We assumed that the peak broadening was a consequence of Lf degradation. Therefore, we continued method optimization using all columns to achieve the separation between Lf and its degradation product(s). During the first method development step, the BioZen™ Intact XB-C8 column proved to be the most promising column regarding the Lf peak shape (Figure A4). It was also the only column that indicated the feasibility of separating Lf from its degradation products.

**Figure A5:**
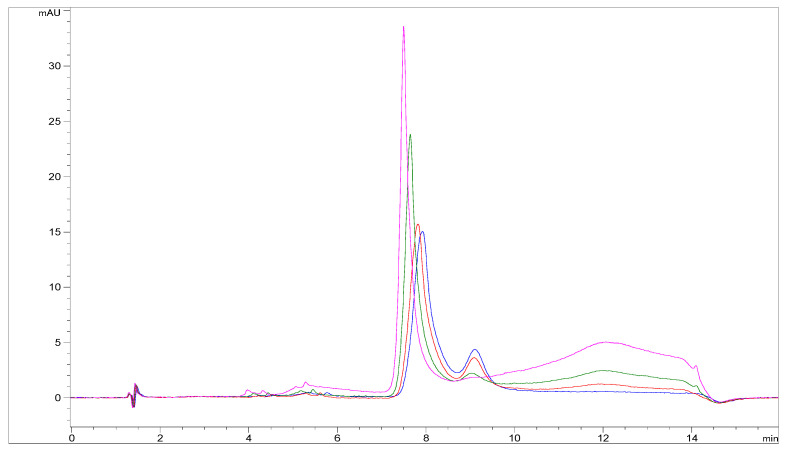
The effect of column temperature on the RP-HPLC chromatograms: −− 30 °C, −− 40 °C, −− 50 °C, and −− 60 °C. Separation was performed on BioZen™ Intact XB-C8 column using 0.1% TFA in water (A) and 0.1% TFA in acetonitrile (B) as a mobile phase at a flow rate of 1.0 mL/min. The gradient program is described in Section 2.2.

Therefore, the BioZen™ Intact XB-C8 column was selected for further optimization in the third method development step. We thoroughly investigated various chromatographic parameters and their corresponding effects on the separation between Lf and its degradation products. The flow rate (0.6–1.2 mL/min) had no significant influence on the chromatographic result. We also tested a wide range of column temperatures (30–60 °C). An increase in column temperature improved the Lf peak shape. However, with the use of higher column temperatures, a decrease in one Lf degradation product peak occurred, which completely disappeared at temperatures above 50 °C (Figure A5). As also lower temperatures prolong the lifetime of a column, the column thermostat was set to 30 °C. Numerous gradient programs were tested and even small changes in gradient programs significantly affected the separation between Lf and its degradation products. The optimal results were achieved on a BioZen™ Intact XB-C8 column at 30 °C by using 0.1% TFA in water and 0.1% TFA in ACN as a mobile phase at a flow rate of 1.0 mL/min. The optimized gradient program was shallow (for more details see Section 2.2) and the final method had a run time of 16 min.

**Figure A6:**
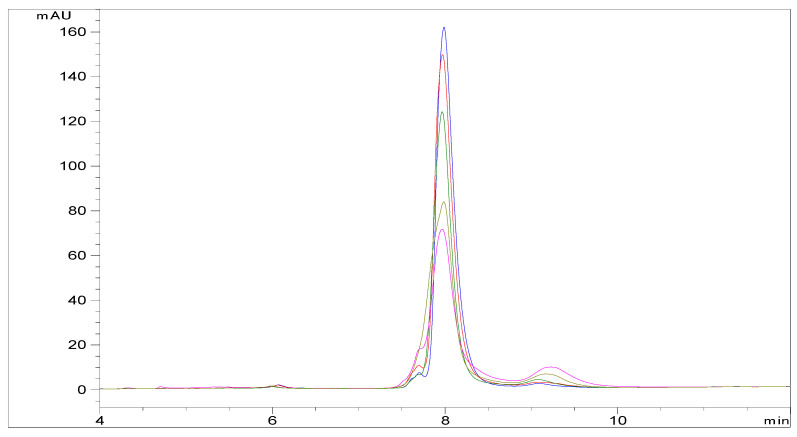
RP-HPLC chromatograms of Lf samples (optimized RP-HPLC method on a BioZen™ Intact XB-C8 column): −− control sample (thermal), −− thermal stress sample (T = 60 °C, *t* = 30 min), −− control sample (acidic and alkaline), −− acidic stress sample (0.1 M HCl, *t* = 3 days), and −− alkaline stress sample (0.01 M NaOH, *t* = 4 h).

Chromatograms of Lf stress samples confirmed the stability-indicating nature of the optimized RP-HPLC method on the BioZen™ Intact XB-C8 column (Figure A6). The forced degradation study showed that Lf is most prone to degradation under alkaline and thermal conditions. About 10% and 20% of Lf degraded after 30 and 60 min at 60 °C, respectively. Lf was extremely unstable in 0.1 M NaOH since about half of Lf degraded after 5 min. Thus, the 0.01 M NaOH was used as an alkaline stress condition. Lf was at least affected by exposure to light as <5% of Lf degraded after one week at daylight.

### 3.2. Size-Exclusion HPLC Method Development and Optimization

Two SEC-HPLC columns that differed in the range of molecular weight distribution, dimensions, and particle size were tested. Phosphate buffers of various pH values and ionic strengths were used as a mobile phase. The effects of mobile phase pH, salt concentration, flow rate, and column temperature on the chromatograms were systematically investigated for an XBridge^®^ Protein BEH SEC 200 Ǻ column. At pH 5.0 and 6.8, the chromatograms were comparable but changed significantly at lower pH making it inadequate for the application. Increasing the ionic strength of the mobile phase (various combinations of different concentrations of phosphate buffer (0.05, 0.1, and 0.2 M) and NaCl (0.0, 0.1, and 0.25 M)) improved the peak shape. Moreover, the stabilization of the chromatographic system was faster at higher ionic strengths of the mobile phase. Column temperature (15–45 °C) had an insignificant impact on chromatographic behavior.

**Figure A7:**
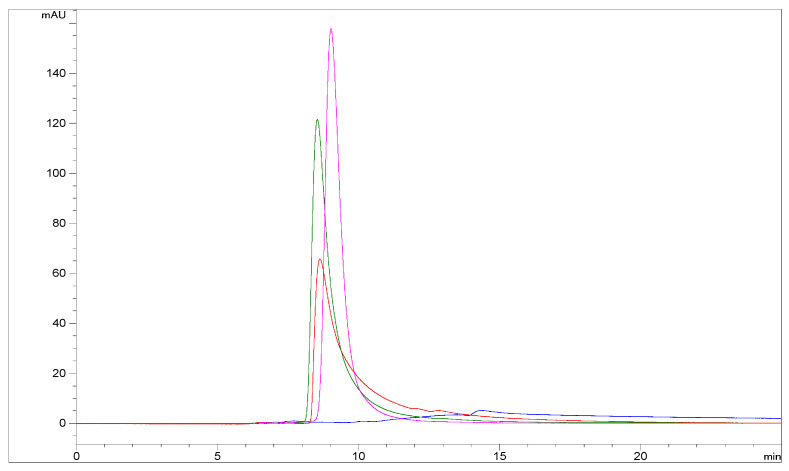
The effect of NaCl concentration in the mobile phase on LF peak shape (SEC column BioSep™-SEC-S 2000): −− 0.0 M NaCl, −− 0.15 M NaCl, −− 0.3 M NaCl, and −− 0.5 M NaCl. Separation was performed on a BioSep™-SEC-S 2000 column at 25 °C using 0.05 M sodium phosphate buffer containing NaCl (pH 7.0) as a mobile phase at a flow rate of 0.8 mL/min).

The effect of mobile phase composition on the Lf peak shape was also evaluated using a BioSep™-SEC-S 2000 column. Changes in mobile phase pH between 6.0 and 7.0 had insignificant impact on the result. Increasing the NaCl concentration in the mobile phase (0.5 M versus 0.0, 0.15, and 0.3 M) improved the Lf peak shape and method sensitivity (Figure A7).

**Figure A8:**
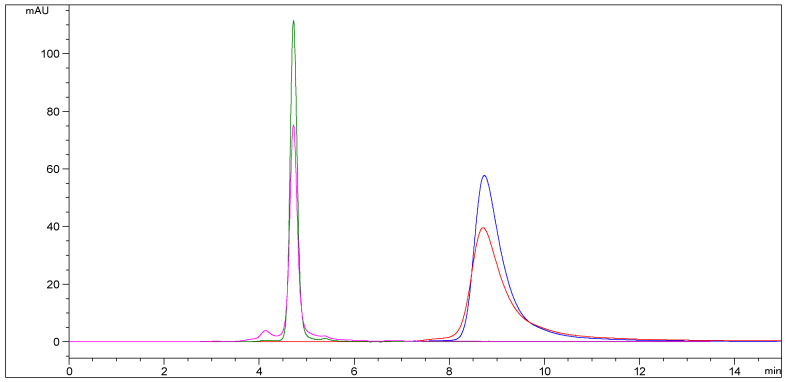
Representative chromatograms of Lf control sample and alkaline stress sample for two SEC columns: −− control sample and −− alkaline stress sample (Separation was performed on a BioSep™-SEC-S 2000 column at 25 °C using 0.05 M sodium phosphate buffer containing 0.5 M NaCl (pH 7.0) as a mobile phase at a flow rate of 0.8 mL/min), −− control sample and −− alkaline stress sample (Separation was performed on an XBridge^®^ Protein BEH SEC 200 Ǻ column at 25 °C using a 0.2 M sodium phosphate buffer containing 0.1 M NaCl (pH 6.8) as a mobile phase at a flow rate of 0.86 mL/min).

**Figure A9:**
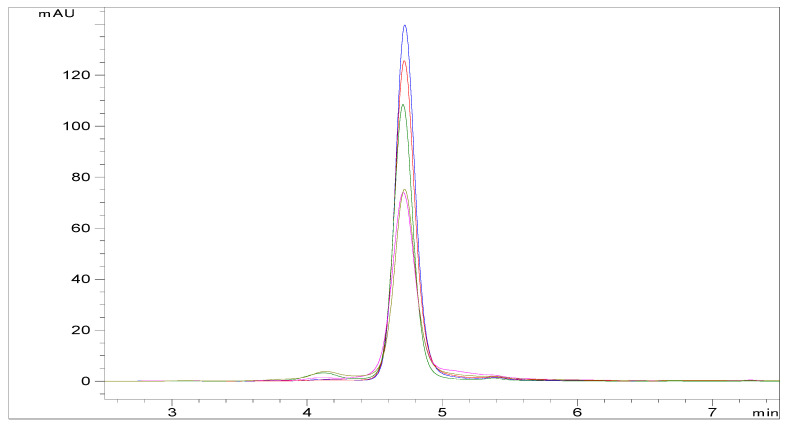
SEC-HPLC chromatograms of Lf samples (optimized SEC-HPLC method on an XBridge^®^ Protein BEH SEC 200 Ǻ column): −− control sample (thermal), −− thermal stress sample (T = 60 °C, *t* = 30 min), −− control sample (acidic and alkaline), −− acidic stress sample (0.1 M HCl, *t* = 3 days), and −− alkaline stress sample (0.01 M NaOH, *t* = 4 h).

At the end, the best results were shown by the XBridge Protein BEH 200 Ǻ column at 25 °C using 0.2 M sodium phosphate buffer (pH 6.8) containing 0.1 M NaCl as a mobile phase at a flow rate of 0.86 mL/min. The Lf peak was sharper and resolved from its degradation products compared to the BioSep™-SEC-S 2000 column where the degradation products could not be observed (Figure A8). In Lf stress samples, peaks representing aggregates and fragments of Lf appeared in chromatograms confirming its stability-indicating nature (Figure A9). They were separated from the main peak. Additionally, the XBridge^®^ Protein BEH SEC 200 Ǻ column is shorter which means shorter equilibration, cleaning, and run times.

### 3.3. Method Validation

The optimized RP-HPLC (BioZen™ Intact XB-C8 column) and SEC-HPLC (XBridge^®^ Protein BEH SEC 200 Ǻ column) methods were successfully validated according to the ICH Q2 (R1) guidelines [26].

#### 3.3.1. Specificity

The specificity was evaluated by comparing the chromatograms of the Lf isolate solution, the compounds used in the sample preparation, and the standard solutions of the most common whey proteins. The developed RP-HPLC method is selective as there were no interfering peaks at the retention time of Lf. For the SEC-HPLC method, the α-lactalbumin and β-lactoglobulin peaks were separated from the Lf peak. However, the lactoperoxidase peak was not separated due to the similar molecular weight of both proteins. Additionally, the specificity of SEC-HPLC and RP-HPLC methods was confirmed by the analyses of commercial products with Lf. There was no co-elution of active ingredients and excipients with the Lf peak. Both methods were also found selective in the presence of degradation products (Figure A6 and Figure A9).

#### 3.3.2. Calibration Curve and Sensitivity

The calibration standards were fitted to different models—linear, weighted linear, and quadratic. The best correlation based on the least-square regression showed the quadratic regression model (Table 2) indicating a certain curvature of the data. The determination coefficient was higher than specified (R^2^ > 0.999) for both chromatographic methods. The calibration curve accuracy for the quadratic regression model showed the best results for the RP-HPLC method (Table 2). It was also the most appropriate model for the SEC-HPLC method but only at Lf concentrations above 0.25 mg/mL. Its suitability with sufficient accuracy was additionally proven using the QC samples (Table 3).

LOD and LOQ results (Table 3) indicate better sensitivity of the RP-HPLC method. The calculated LOD and LOQ values are below the concentration of the lowest calibration standard for both methods. The proposed analytical range (0.05–5.0 mg/mL) can be applied by using the RP-HPLC method. However, from practical experience, it is recommended that the concentration of Lf in the samples is above 0.15 mg/mL by using the SEC-HPLC method as confirmed by QC_l_ samples (Table 3).

#### 3.3.3. Accuracy and Precision

The intra- and inter-day accuracy and precision results are summarized in Table 3. The results for both methods were following the defined criteria. The only slight deviation was the inter-day precision at QC_l_ for the SEC-HPLC method. Based on the validation results (Table 2 and Table 3), the RP-HPLC method is more appropriate for the quantitative analysis of Lf samples. The adequacy of the RP HPLC method for quantification purposes was additionally confirmed by method recovery determination, which was within 95 and 105% for all analyzed Lf samples (Table 4).

#### 3.3.4. Sample Stability

The sample stability in the autosampler at 25 °C after 24 and 48 h was evaluated using the RP-HPLC method due to its better accuracy and precision results. The stability of Lf in quality control samples at three concentration levels (0.15, 0.75, and 4.0 mg/mL) was within the defined acceptance interval (100 ± 5%) at both time points.

### 3.4. Comparison with Spectroscopic Techniques

**Figure A10:**
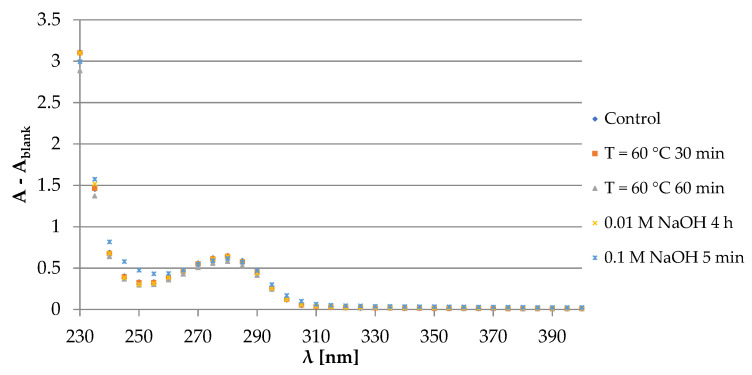
Absorption spectra of various Lf stress samples compared to Lf control sample (A_280nm_ method).

**Figure A11:**
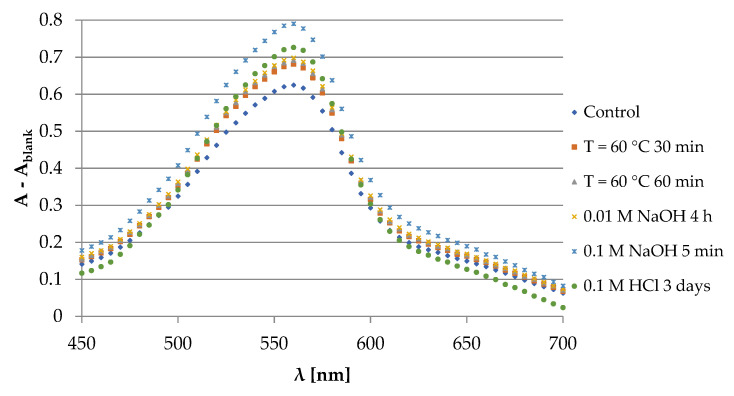
Absorption spectra of various Lf stress samples compared to Lf control sample (BCA method).

**Figure A12:**
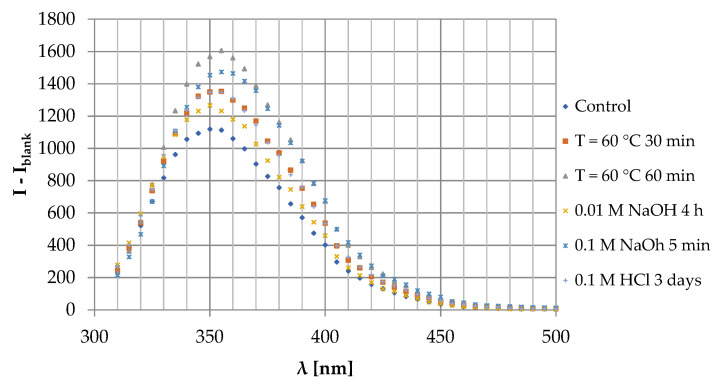
Emission spectra of various Lf stress samples compared to Lf control sample (FLD method).

Three spectroscopic methods (A_280nm_, FLD, and BCA) were included to evaluate their potential use in Lf stability studies and for the determination of its content in commercially available products. Lf stress samples were used to evaluate their specificity and stability-indicating nature. Stress samples in oxidative medium interfered with all three methods. There were no changes in Lf signal intensity when measuring absorbance at 280 nm in other stress samples compared to the control sample (Figure A10). However, by using BCA and FLD methods, the signal intensity was significantly higher for stress samples (Figure A11 and Figure A12). There was also a slight change in the FLD spectrum (emission maximum shifted from 350 to 355 nm) of Lf stress samples (Figure A12).

Validation of spectroscopic methods was performed to a lesser extent compared to chromatographic methods. The results are presented in Table 4. The determination coefficients were high (R^2^ > 0.999) for all three spectroscopic methods. The quadratic regression was also the best fitting model for spectroscopic methods. The accuracy and precision of the FLD method strongly deviated from the specified values making it inappropriate for quantitative analysis. The precision and accuracy results were better for the A_280nm_ method than for the BCA method (Table 4). Moreover, the denaturation of Lf could result in a falsely higher content determination by using the BCA method. Validation and method recovery results indicate that only the A_280nm_ method could be used for quantification purposes (Table 4 and Table 5). However, due to lack of selectivity, it is only valuable for the samples that do not contain any additional chromophores which absorb at 280 nm. In comparison, better selectivity of the RP-HPLC method allows the determination of Lf content in complex samples. Chromatographic methods are also appropriate for qualitative and quantitative evaluation of Lf stability as they are capable of discriminating between different Lf stress samples. However, the complementary information from simpler, faster, and more accessible spectroscopic techniques may be valuable in some cases.

### 3.5. Application of Methods

#### 3.5.1. Stability of Lactoferrin at High Temperatures

The optimized stability-indicating analytical approach was applied to demonstrate its usefulness for stability studies and potential formulation purposes. Firstly, the Lf stability at elevated temperatures (50–80 °C) was evaluated as the temperature is one of the key factors affecting the Lf stability. The validated RP-HPLC method was used for qualitative as well as quantitative purposes. Additionally, samples were analyzed by the SEC-HPLC method to obtain complementary qualitative data about the degradation of Lf, especially to detect potentially formed aggregates or fragments of Lf.

**Figure 1:**
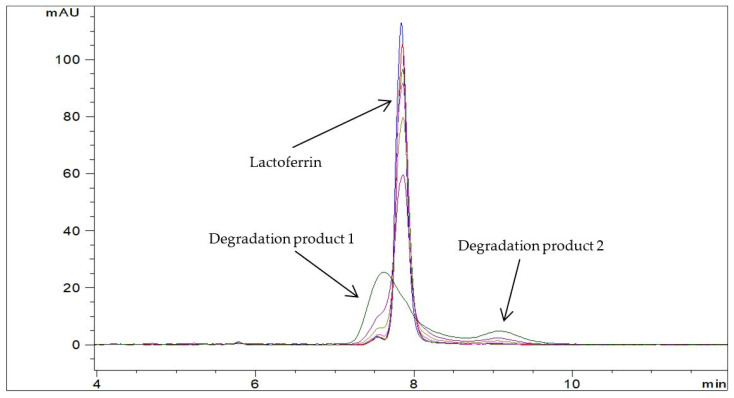
Representative chromatograms of solid Lf isolate sample 1 in Milli-Q water (c = 1.0 mg/mL) at 50 °C at different time points: −− t = 0 min, −− t = 15 min, −− t = 30 min, −− t = 60 min, −− t = 120 min, −− t = 240 min, and −− t = 24 h (RP-HPLC method).

**Figure 2:**
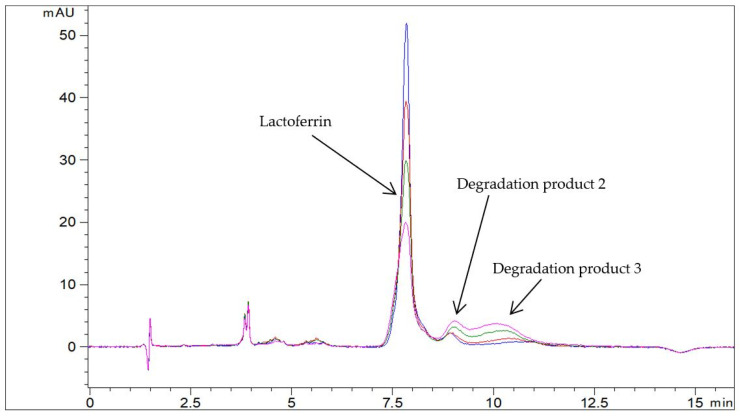
Representative chromatograms of Jarrow Formulas capsules with Lf in Milli-Q water (c = 1.0 mg/mL) at 60 °C at different time points: −− t = 0 min, −− t = 15 min, −− t = 30 min, and −− t = 60 min (RP-HPLC method).

**Figure 3:**
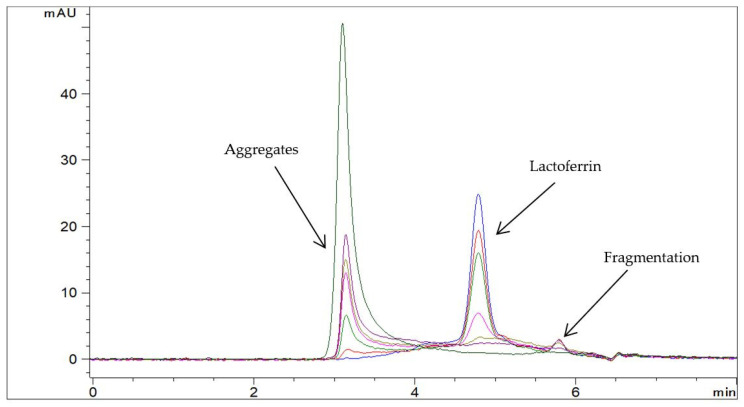
Representative chromatograms of Jarrow Formulas capsules with Lf in Milli-Q water (c = 1.0 mg/mL) at 60 °C at different time points: −− t = 0 min, −− t = 15 min, −− t = 30 min, −− t = 60 min, −− t = 120 min, −− t = 240 min, and −− t = 24 h (SEC-HPLC method).

Two degradation products were detected in aqueous Lf isolate samples at elevated temperatures by the RP-HPLC method (Figure 1). Degradation product 1 eluted in front of Lf whereas degradation product 2 eluted approximately 1 min after Lf. The profile of degradation products at different storage temperatures and from different lots of the Lf isolate samples was the same. In a sample of Jarrow Formulas capsules containing Lf at elevated temperature, the profile of degradation products was altered. An additional degradation product with a longer retention time (degradation product 3) was detected (Figure 2). In both cases, the Lf peak was gradually decreasing, and the peaks for degradation products were increasing over time. SEC-HPLC analysis also revealed some differences in degradation mechanisms of both samples. Aggregates and fragments of Lf were observed in a sample of capsules at elevated temperature (Figure 3). For the Lf isolate sample, no aggregates were observed at 60 °C; however, they were formed at higher temperatures (70 and 80 °C). After longer exposure to elevated temperatures, Lf in this sample precipitated into larger particles resulting in opaque sample solutions.

The validated RP-HPLC method was also used for the quantitative evaluation of the Lf stability at higher temperatures. The fitting of several kinetic models to Lf degradation at various temperatures is shown in Table 6. The determination coefficients were the highest for the zero-order kinetic model for three temperatures. Therefore, it was used for the determination of rate constants. About 80% of initial Lf content remained in the Lf isolate sample 1 after 4 h at 50 °C, while Lf totally degraded in less than 1 h at 80 °C. In samples of Jarrow Formulas capsules exposed to high temperature (60 °C), Lf degraded faster compared to the Lf isolate sample 1 at the same temperature. The degradation was approximately as fast as for the Lf isolate sample 1 at 70 °C. Stability differences were also observed between different lots of Lf isolate samples. Three lots of Lf isolate samples with different characteristics (Table A1) but similar iron saturation were kept at 50 °C and the calculated zero-order rate constants for the degradation of Lf were 0.0487, 0.0376, and 0.0549 mg mL^−1^·h^−1^, respectively.

**Figure 4:**
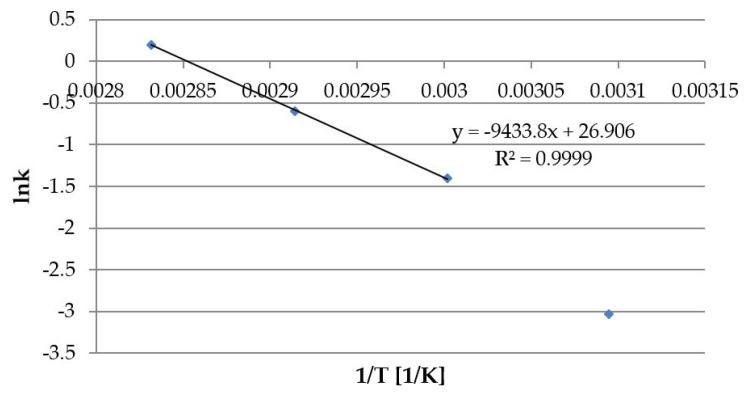
Arrhenius plot for solid Lf isolate sample 1 (c_0_ = 1.0 mg/mL) based on zero-order rate constants for the degradation of Lf at 50–80 °C.

The Arrhenius plot was constructed to correlate storage temperature and stability of Lf (Figure 4). The Arrhenius equation was linear in the temperature range from 60 to 80 °C. The determination coefficient was high (>0.999). However, at 50 °C the rate constant deviated from the correlation. This may be due to the changed degradation mechanism of Lf in the temperature range between 50 and 60 °C. The activation energy calculated from the higher temperature range was found to be 78.4 kJ/mol.

#### 3.5.2. Stability of Lactoferrin in Elution Samples

Due to observed differences in stability of various Lf samples, the analytical approach was extended to other samples from different stages of Lf isolation. The aim was to further investigate storage conditions, appropriate for the storage of these samples. Lf in the E3 sample was stable at least 1 month at 4 °C and −20 °C (Table 7), while its concentration decreased by 11.6% after 1 month at room temperature. The stability of Lf in the concentrated E3 sample was lower than in the E3 sample. The content of Lf in concentrated E3 sample dropped to about 90% of the initial content after 2 weeks at all storage temperatures. The degradation was the fastest at room temperature (Table 7). Due to different elution conditions, the E1 sample was significantly less pure and contained also other proteins besides Lf (Table A1). The degradation of Lf in the E1 sample was several-fold faster compared to E3 and concentrated E3 samples (Table 7).

Since the stability of Lf in E1 (0.55 mg/mL), E3 (1.9 mg/mL) and concentrated E3 sample (30.5 mg/mL) were not comparable, a stability experiment was conducted to evaluate the effect of concentration of Lf on its stability. Samples were relatively stable for four weeks at 25 °C as only about 10% of initial Lf was degraded. No significant differences in rate constants between samples of different Lf concentrations were observed (Table 7). From these results, it could be concluded that the Lf concentration is not the reason for Lf stability differences in E1, E3, and concentrated E3 samples.

## 4. Discussion

Lf, as well as other proteins, are structurally much more complex than small molecules. Therefore, they demand a complex analytical approach to be used in stability studies. We aimed to develop methods that could separate Lf from its degradation products and thus be used for stability evaluation of Lf, as well as for quality control of products containing Lf. Lf stress samples were used for the development of a stability-indicating analytical approach which was comprised of two complementary techniques: RP-HPLC and SEC-HPLC. To the best of our knowledge, the forced degradation approach has not been applied earlier for the evaluation of Lf stability. The RP-HPLC method development was more challenging due to the complexity of the separation mechanism and specific characteristics of protein molecules.

The column selection is one of the key parameters in HPLC method development. Therefore, four RP-HPLC columns with expected differences in their selectivity to ensure different interactions with Lf were tested. Firstly, the Lf peak shape was optimized on each column. The combination of TFA in water and ACN was selected for the gradient elution of Lf as it is a commonly used mobile phase in protein analysis. A higher concentration of ion-pairing agent resulted in a sharper chromatographic peak of Lf. The addition of NaCl to the mobile phase, which we found in some of the published RP-HPLC methods for Lf [8,27], was crucial only for the older type of traditional porous column. The lowest tested NaCl concentration (0.05 M) was sufficient for an improved peak shape. This is an improvement compared to the published methods that use a high NaCl concentration (0.5 M) [8,27]. A high salt concentration is undesirable because salt can precipitate and cause blockages, especially with a higher percentage of organic solvents. For other tested RP-HPLC columns the addition of NaCl was non-essential or even worsened the Lf peak shape. The gradient program was also optimized for each column to obtain sharp Lf peaks. It was noticed that the percent of organic solvent needed for the elution of Lf was independent of the stationary phase chemistry (C3–C18). In the second method development step, the forced degradation approach was introduced as it is the key to stability-indicating method development. The Lf chromatographic peak in stress samples broadened regardless of the column used which indicated the formation of degradation products (Figure A4). Therefore, additional efforts were made to achieve the separation between Lf and its potential degradation products. The only column capable of separating Lf from its degradation products was the BioZen™ Intact XB-C8 column. A shallow gradient proved to be the most appropriate for the separation between Lf and its degradation products. It was also noticed that small changes in gradient program significantly affect the chromatographic behavior which is also common for other protein analysis [28]. The final method with the run time of 16 min is shorter than many of the published RP-HPLC non-stability-indicating methods for Lf [8,11,15,16,17].

The SEC-HPLC is a commonly used technique in protein analysis. It is valuable for the determination of protein aggregation which is a common type of protein instability [29,30]. The selection of the column and the mobile phase are the most important chromatographic parameters. The composition of the mobile phase had a significant effect on the Lf peak shape. Increasing the ionic strength of the mobile phase improved the method sensitivity on both tested columns. Higher ionic strength also solved the issue of slower chromatographic system stabilization observed with an XBridge^®^ Protein BEH SEC 200 Ǻ column. A high salt concentration reduces the secondary electrostatic interactions between the analyte and stationary phase and leads to a better peak symmetry and improves quantification [30]. Based on the forced degradation samples, the XBridge^®^ Protein BEH SEC 200 Ǻ column was selected as the Lf peak was sharper and resolved from its degradation products (Figure A8).

The forced degradation study showed on both chromatographic methods that Lf was most prone to degradation under alkaline and thermal conditions and at least affected by exposure to light. The developed stability-indicating approach was successfully validated according to the ICH guidelines, confirming their precision, accuracy, and adequacy of the quadratic regression model. Both methods were selective in the presence of Lf degradation products confirming their stability-indicating nature and adequacy for the Lf stability studies. Based on the validation results (Table 2 and Table 3), the SEC-HPLC method is less appropriate for the quantitative analysis of Lf samples. However, it is a valuable support and complement to the RP-HPLC method in stability testing.

Our analytical approach was additionally extended with three spectroscopic techniques to investigate their potential use in Lf stability studies. By using BCA and FLD methods, the signal intensity was significantly higher for the Lf stress samples compared to control samples. Moreover, the emission maximum shifted from 350 to 355 nm in the FLD spectrum, which is typical for tryptophan residues in unfolded proteins [20]. The BCA and FLD methods could be used for qualitative evaluation of the Lf stability and consequently for assessing the changes in coiling of the protein [19,20,31]. On the other hand, the A_280nm_ method was not appropriate for the Lf stability evaluation. However, the validation parameters for the A_280nm_ method showed the best performance among the tested spectroscopic methods. Real samples analysis revealed that precision and recovery of the A_280nm_ method is acceptable, however, it lacks selectivity. Therefore, this method could be used for the quantification of Lf in simple matrices without interferences of other compounds at 280 nm (Table 5 and Table A1). Analysis of commercial products containing Lf by the RP-HPLC method confirmed its suitable method performance including the determined contents of Lf, which were within 25% of the declared contents. This is following our expectations because food supplements are not strictly regulated. Lf content in food supplements was assumed to be in close agreement to regulated active components such as vitamins, which contents have been found even with higher deviations from the label claims [32], confirming that the RP-HPLC method is appropriate for quality control of products containing Lf.

To demonstrate the applicability of the developed analytical approach, it was used in preliminary stability studies. Three degradation products were detected in various aqueous Lf samples by RP-HPLC. To the best of our knowledge, this is the first RP-HPLC method capable of detecting Lf degradation products. Other researchers had similar intentions, but they did not manage to develop such methods [8,17]. Valuable complementary information by SEC-HPLC analyses also revealed some differences in degradation mechanisms between different Lf samples. The differences in aggregation behavior of Lf in these samples could be due to different glycosylation patterns or salt concentrations. Salt causes the reduction in electrostatic repulsion between the individual proteins which promotes the formation of large particles [33]. This could be the reason for opaque sample solutions of Lf isolate samples at elevated temperatures since salts were used in the isolation process. The profile of degradation products, as well as the kinetics of Lf degradation, was different in various aqueous Lf samples (see Figure 1, Figure 2 and Figure 3), confirming the adequacy of the introduced analytical approach.

The degradation of Lf at elevated temperatures was also quantitatively evaluated using the RP-HPLC method. The degradation of Lf in aqueous solution at 50–80 °C best fitted the zero-order kinetic model (Table 6) This is not in line with literature data that report either first or 1.5 order kinetics [34,35,36]. However, the determination coefficients (R^2^) obtained in our study for the zero-order model were higher compared to those found in other research articles. With our analytical methodology it was possible to observe different degradation rates in different Lf samples. A correlation between storage temperature and degradation of Lf was also observed. The Arrhenius equation was linear in the temperature range from 60 to 80 °C. However, the rate constant at 50 °C significantly deviated from the correlation (Figure 4) indicating changes in the degradation mechanism of Lf between 50 and 60 °C. Our findings are following results obtained by other researchers who used the same Lf isolate samples to study the thermodynamic stability by UV–VIS spectroscopy. It was found that Lf is thermally stable up to about 53 °C with a denaturation temperature of 58.6 °C [21]. The activation energy calculated from the Arrhenius plot was found to be 78.4 kJ/mol, which is lower than values found in the literature. However, a completely different analytical technique was used in these studies [34,35,36]. The differences could also be due to different iron saturation and glycosylation patterns of Lf and/or media used in stability studies [12].

We extended the stability testing of Lf at real storage conditions. Using our stability-indicating approach we found out that Lf was significantly more stable at real storage conditions compared to temperatures above 50 °C with rate constants more than two orders of magnitude lower at real storage conditions. The samples were predictably less stable at room temperature than in the refrigerator or in the freezer (Table 7). However, significant differences in Lf stability among various elution samples, which differed in Lf concentration and its purity (Table A1), were observed. This is most likely due to the presence of other protein impurities or different salt concentrations as known from the literature [12,33] since samples with various concentrations of the same Lf did not affect Lf rate constants (Table 7).

## 5. Conclusions

Proteins are complex macromolecules and consequently the evaluation of their stability demands a complex analytical approach. Herein, an alternative analytical approach for stability evaluation of lactoferrin is proposed, in which the forced degradation approach was applied for the first time. The developed stability-indicating analytical approach is comprised of complementary reversed-phase and size-exclusion HPLC methods. The reversed-phase HPLC method is appropriate for quantitative as well as qualitative analyses, whereas important complementary qualitative information about aggregation and fragmentation of lactoferrin can be obtained using the size-exclusion HPLC method. The described HPLC methods are favorable compared to other published methods because of their stability-indicating nature, the ability to detect different degradation products, and short-run times, while also having a relatively wide analytical range, good accuracy, and precision. The analytical approach was additionally extended to commonly used spectroscopic techniques. They proved to be less appropriate for qualitative and quantitative evaluation of lactoferrin than chromatographic methods. However, the complementary information from simpler, faster, and more accessible spectroscopic techniques may be valuable in some cases. The applicability of the developed approach was demonstrated in several preliminary stability experiments. Up to three degradation products, as well as aggregates and fragments of lactoferrin, were detected in various samples by using both, reversed-phase HPLC and size-exclusion HPLC methods. The presented analytical approach may be used for the quantitative evaluation of lactoferrin stability in various samples including the ability to detect differences in degradation mechanisms. The differences in stability of lactoferrin in various samples were observed and derived conclusions are in line with literature data. The developed analytical approach has also the potential to be used in the quality control of lactoferrin products. However, the methods described herein may not be directly assimilated to the biological activities of lactoferrin.

## Figures and Tables

**Table 1:** Preparation of lactoferrin (Lf) stress samples.

Condition	Preparation ^a^	Time	Sample Treatment before Analysis
Control: Milli-Q water Room T	1000 µL Lf sample	/	/
Thermal: Milli-Q water Water bath, T = 60 °C	1000 µL Lf sample	*t*_1_ = 30 min *t*_2_ = 60 min	Cooldown to room T
Alkaline: 0.01 M NaOH ^b^ Room T	900 µL Lf sample + 100 µL 0.1 M NaOH	*t*_1_ = 4 h	Addition of 100 µL 0.1 M HCl
Acidic: 0.1 M HCl Room T	900 µL Lf sample + 100 µL 1 M HCl	*t*_1_ = 3 days	Addition of 100 µL 1 M NaOH
Oxidative: 3% H_2_O_2_ Room T	900 µL Lf sample + 100 µL 30% H_2_O_2_	*t*_1_ = about 24 h	/
Light: Milli-Q water Room T, daylight	1000 µL Lf sample	*t*_1_ = 7 days	/

^a^ Concentration of Lf in the sample was 1.0 mg/mL; ^b^ the degradation of Lf was too fast in 0.1 M NaOH.

**Table 2:** Comparison of calibration curve models for reversed-phase (RP-HPLC) and size-exclusion (SEC-HPLC) methods.

Methods	Models	R^2^	Calibration Curve Accuracy [%]							
			0.05	0.10	0.25	0.50	1.0	1.75	2.5	5.0
RP-HPLC	Linear	0.9985	201.0	141.8	111.8	99.6	96.7	97.4	95.1	101.6
	Weighted linear (1/c)	0.9981	115.2	100.3	97.0	93.7	95.3	97.9	96.4	104.1
	Weighted linear (1/c^2^)	0.9977	102.9	95.9	97.6	95.8	98.3	101.4	100.0	108.1
	Quadratic	0.9999	96.0	94.9	100.2	98.9	100.6	101.9	98.7	100.1
SEC-HPLC	Linear	0.9995	200.1	138.4	103.1	96.5	94.5	97.8	99.2	100.7
	Weighted linear (1/c)	0.9980	123.3	101.1	89.6	91.1	93.1	98.4	100.5	102.9
	Weighted linear (1/c^2^)	0.9937	105.2	94.5	90.0	94.2	97.6	103.7	106.1	108.9
	Quadratic	0.9999	149.2	115.4	96.9	96.0	96.4	100.2	101.0	99.9

**Table 3:** Validation data for RP-HPLC and SEC-HPLC methods.

Validation Parameters			SEC-HPLC	RP-HPLC
Range [mg/mL]			0.05–5.0	0.05–5.0
Determination coefficient (R^2^)			0.9999	0.9999
LOD [mg/mL]			0.007	0.001
LOQ [mg/mL]			0.022	0.004
Accuracy [%]	Intra-day	QC_l_	102.3	96.4
		QC_m_	99.2	99.8
		QC_h_	98.4	102.3
	Inter-day	QC_l_	100.1	95.4
		QC_m_	94.9	99.9
		QC_h_	99.0	102.0
Precision [%]	Intra-day	QC_l_	0.9	1.5
		QC_m_	1.8	2.7
		QC_h_	0.6	0.9
	Inter-day	QC_l_	6.1	2.9
		QC_m_	3.5	2.4
		QC_h_	0.9	0.8
	Injection repeatability	QC_m_	1.3	0.2

**Table 4:** Validation data for spectroscopic methods.

Validation Parameters			A_280nm_	BCA	FLD
Range [mg/L]			0.05–5.0	0.025–2.0	0.005–0.500
Determination coefficient (R^2^)			0.9999	0.9993	0.9990
Accuracy [%]	Intra-day	QC_l_	100.3	94.4	115.2
		QC_m_	102.1	106.5	94.5
		QC_h_	107.6	96.0	92.4
Precision [%]	Intra-day	QC_l_	4.7	11.6	32.0
		QC_m_	0.01	2.7	17.2
		QC_h_	2.3	2.9	3.4

A_280nm_: measurements of absorbance at 280 nm; BCA: the bicinchoninic acid method for total protein concentration determination; FLD: intrinsic fluorescence measurements.

**Table 5:** Analysis of samples containing lactoferrin.

Real Samples Analysis		RP-HPLC	A_280nm_	BCA
Capsules Jarrow Formulas	Content [%] ^a^	75.4 ± 1.9	86.4 ± 2.1	100.5 ± 7.7
	Recovery [%]	96.3 ± 3.3	102.2 ± 5.7	89.3 ± 19.6
Capsules Sensilab	Content [%] ^a^	96.0 ± 6.0	201.9 ± 5.7	89.0 ± 3.3
	Recovery [%]	96.6 ± 1.4	108.5 ± 4.8	78.8 ± 12.8
Capsules Peau radieuse	Content [%] ^a^	124.2 ± 4.5	589.2 ± 25.6	265.3 ± 11.8
	Recovery [%]	101.8 ± 1.6	100.5 ± 2.4	80.8 ± 2.5

^a^ Ratio between the determined and the labeled content of Lf expressed as a percentage.

**Table 6:** Kinetic models for Lf degradation along with corresponding determination coefficients (R^2^) under various temperatures.

T [°C]		R^2^	
	0. Order	1. Order	2. Order
50	0.9923	0.9916	0.9895
60	0.9796	0.9532	0.9185
70	0.9911	0.9956	0.9654
80	0.9980	0.9851	0.9288

**Table 7:** Degradation rate constants for various Lf samples at different temperatures.

Sample	k [% Day^−1^] ^a^		
	Room T	4 °C	−20 °C
E1 sample (c = 0.55 mg/mL)	5.585	/	/
E3 sample (c = 1.9 mg/mL)	0.438	Stable ^b^	Stable ^b^
Concentrated E3 sample (c = 30.5 mg/mL)	1.302	0.529	0.814
Lf isolate sample 3 (c = 0.55 mg/mL)	0.326	/	/
Lf isolate sample 3 (c = 1.9 mg/mL)	0.353	/	/
Lf isolate sample 3 (c = 10.0 mg/mL)	0.409	/	/
Lf isolate sample 3 (c = 30.5 mg/mL)	0.426	/	/

^a^ Zero-order degradation rate constant. Content of Lf [%] was calculated relatively to the zero-time point and used for the calculation of rate constants to obtain comparable results due to the high concentration difference. ^b^ <5% Lf degradation after 4 weeks; / not evaluated.

## Data Availability

Data are contained within the article.

## Appendix A

**Table A1:** Extended data of tested products containing lactoferrin.

Product, Manufacturer	Dosage Form	Labeled Content of Lf	Other Ingredients	
Lactoferrin, Jarrow Formulas^®^ (Los Angeles, CA, USA)	Capsule	250 mg	cellulose, magnesium stearate, silicon dioxide gelatin (capsule shell)	
Cerin^®^ Acne Treatment, Sensilab^®^ (Ljubljana, Slovenia)	Capsule	100 mg	zinc gluconate, horsetail aerial part extract with 7% silica, wild pansy aerial part extract, bitter orange extract with 98% hesperidin, D-biotin 1% on maltodextrin, retinyl palmitate, copper citrate, magnesium stearate, black pepper fruit extract with 95% piperine, riboflavin, thiamine hydrochloride, hydroxypropyl methylcellulose (capsule shell), titanium dioxide, yellow iron oxide	
Peau radieuse^®^, Laboratoire Biocyte (Mougins, France)	Capsule	20.5 mg	methylsulfonylmethane, greater burdock extract, flowering bugleweed extract, common nettle leaf powder, yeast extract including beta-glucans, maltodextrin, magnesium salts of fatty acids, zinc oxide, silicon dioxide, chromium picolinate, gelatin (capsule shell), titanium dioxide	
**Product,** **manufacturer**			**Concentration of Lf**	**Chromatographic purity [%] ^a^**
Liquid Lf isolate: E1 sample, ARHEL d.o.o.			0.55 mg/mL	27.5
Liquid Lf isolate: E3 sample, ARHEL d.o.o.			1.9 mg/mL	91.7
Liquid Lf isolate: concentrated E3 sample, ARHEL d.o.o.			30.5 mg/mL	96.0
**Product,** **manufacturer**			**Content of Lf [m/m %]**	**Chromatographic purity [%] ^a^**
Solid Lf isolate sample 1, ARHEL d.o.o.			93.4	97.1
Solid Lf isolate sample 2, ARHEL d.o.o.			86.1	85.4
Solid Lf isolate sample 3, ARHEL d.o.o.			88.1	86.9

^a^ Chromatographic purity was assessed by the proposed RP-HPLC method.
